# Child and Adolescent Depression: A Review of Theories, Evaluation Instruments, Prevention Programs, and Treatments

**DOI:** 10.3389/fpsyg.2019.00543

**Published:** 2019-03-20

**Authors:** Elena Bernaras, Joana Jaureguizar, Maite Garaigordobil

**Affiliations:** ^1^Developmental and Educational Department, University of the Basque Country, Donostia/San Sebastián, Spain; ^2^Developmental and Educational Psychology Department, University of the Basque Country, Lejona, Spain; ^3^Personality, Evaluation and Psychological Treatments Department, University of the Basque Country, Donostia/San Sebastián, Spain

**Keywords:** depression, adolescent, child, instruments, prevention, treatment

## Abstract

Depression is the principal cause of illness and disability in the world. Studies charting the prevalence of depression among children and adolescents report high percentages of youngsters in both groups with depressive symptoms. This review analyzes the construct and explanatory theories of depression and offers a succinct overview of the main evaluation instruments used to measure this disorder in children and adolescents, as well as the prevention programs developed for the school environment and the different types of clinical treatment provided. The analysis reveals that in mental classifications, the child depression construct is no different from the adult one, and that multiple explanatory theories must be taken into account in order to arrive at a full understanding of depression. Consequently, both treatment and prevention should also be multifactorial in nature. Although universal programs may be more appropriate due to their broad scope of application, the results are inconclusive and fail to demonstrate any solid long-term efficacy. In conclusion, we can state that: (1) There are biological factors (such as tryptophan—a building block for serotonin-depletion, for example) which strongly influence the appearance of depressive disorders; (2) Currently, negative interpersonal relations and relations with one's environment, coupled with social-cultural changes, may explain the increase observed in the prevalence of depression; (3) Many instruments can be used to evaluate depression, but it is necessary to continue to adapt tests for diagnosing the condition at an early age; (4) Prevention programs should be developed for and implemented at an early age; and (5) The majority of treatments are becoming increasingly rigorous and effective. Given that initial manifestations of depression may occur from a very early age, further and more in-depth research is required into the biological, psychological and social factors that, in an interrelated manner, may explain the appearance, development, and treatment of depression.

## Introduction

Depression is the principal cause of illness and disability in the world. The World Health Organization (WHO) has been issuing warnings about this pathology for years, given that it affects over 300 million people all over the world and is characterized by a high risk of suicide (the second most common cause of death in those aged between 15 and 29) [World Health Organization (WHO), 2017]. Studies on the child population which use self-reports to evaluate severe symptoms of depression, specifically the Children's Depression Inventory (CDI, Kovacs, 1992) and the Children's Depression Scale (CDS, Lang and Tisher, 1978), have observed prevalence rates of, for example, 4% in Spain (Demir et al., 2011; Bernaras et al., 2013), 6% in Finland (Puura et al., 1997), 8% in Greece (Kleftaras and Didaskalou, 2006), 10% in Australia (McCabe et al., 2011), and 25% in Colombia (Vinaccia et al., 2006). The main classifications of mental disorders are the Diagnostic and Statistical Manual of Mental Disorders, DSM-5 (American Psychiatric Association, 2014), published by the American Psychiatric Association, which has become a key reference in clinical practice, and version 10 of the International Classification of Diseases (ICD-10, 1992), published by the WHO, which classifies and codifies all diseases, although initially its aim was to chart mortality rates. The new ICD-11 classification will be presented for approval to Member States at the World Health Assembly in May 2019, and is expected to come into effect on January 1, 2022 [World Health Organization (WHO), 2018]. The two classifications offer different categorizations of depressive disorders, although certain similarities do exist, and it should be borne in mind also that both have been criticized for hardly distinguishing at all between child and adult depression.

Throughout history, there have been many different explanatory theories of depression. Biological and psychological theories are the ones which have mainly tried to explain the origin of this mental disorder. Biological theories have, from a variety of different perspectives, postulated that depression may occur due to noradrenalin deficits (e.g., Schildkraut, 1965; Narbona, 2014), endocrine disorders (e.g. Birmaher et al., 1996), sleep-related disorders (e.g., Sivertsen et al., 2014; Pariante, 2017), alterations in brain structure (Whittle et al., 2014), or the influence of genetics (Scourfield et al., 2003). Psychological theories have attempted to explain depression on the basis of psychoanalysis and, more specifically, in terms of attachment theories (e.g., Bowlby, 1976; Ainsworth et al., 1978; Blatt, 2004; Bigelow et al., 2018), behavioral models (e.g., Skinner, 1953; Ferster, 1966; Lewinsohn, 1975), cognitive models (e.g., Seligman, 1975; Abramson et al., 1978; Beck, 1987), the self-control model (e.g., Rehm, 1977; Rehm et al., 1979), interpersonal theory (e.g., Markowitz and Weissman, 1995; Milrod et al., 2014), stressful life events (e.g., Reinherz et al., 1993; Frank et al., 1994), and sociocultural models (e.g., Lorenzo-Blanco et al., 2012; Chang et al., 2013; Reeves et al., 2014).

Evaluating depression accurately has been another concern upon which psychology has focused, with attention being centered specifically around diagnosing this pathology in childhood and adolescence. Although many diagnostic instruments have been developed and validated, mainly for the adolescent and adult stages of life, it is still difficult to find diagnostic tests for evaluating depression in children. Preventing depression is another aspect to which much importance is attached by the World Health Organization (WHO) (2017), which argues that school programs, interventions aimed at parents and specific exercises for the elderly population help reduce the prevalence of this pathology. Depression prevention programs do exist, but they are mainly targeted at adolescents and very few focus on children under the age of 10.

The treatment of depression is another aspect that should not be overlooked. In 2016, the WHO and the World Bank announced that investing in the treatment of depression and anxiety leads to four-fold returns, since these pathologies cost the global economy one trillion US dollars each year. Furthermore, they claimed that humanitarian emergencies and conflicts highlight a pressing need to broaden current therapeutic options. In this sense, the multiple different explanatory theories of depression have given rise to a plethora of different treatments (psychotherapeutic, behavioral, cognitive-behavioral, interpersonal, etc.) which are currently being analyzed with a high degree of precision and scientific rigor.

In light of the different aspects related to depression outlined above, the present study has the following aims: (1) To analyze the construct of depression offered by the two main mental disorder classifications (DSM-5 and ICD-10); (2) To provide an overview of the main explanatory theories of depression; (3) To outline the child and adolescent depression evaluation instruments most commonly used in scientific literature; (4) To provide a brief overview of child and adolescent depression prevention programs in the school environment; and (5) To describe the most scientifically rigorous and effective clinical treatments for this mental disorder.

The databases used for carrying out the searches were PubMed, PsycINFO, Web of Science, Scopus, Science Direct and Google Scholar, along with a range of different manuscripts. With the constant key word being depression, the search for information cross-referenced a series of other key words also, namely: childhood, adolescence, explanatory theories, etiology, evaluation instruments, prevention programs, and treatment. Searches were conducted for information published between 1970 and 2017.

Thus, first we describe the construct of depression and summarize the main explanatory theories. Next, we present the main evaluation instruments used to measure child and adolescent depression and report the results of a bibliographical review of prevention programs in school settings. Finally, we outline the main clinical treatments used nowadays to treat child and adolescent depression.

## The Construct of Depression: DSM-5 and ICD-10

Depression features in both of the two most important global classifications: the DSM-5 and the ICD-10. As stated earlier in the introduction, the new ICD-11 classification will be presented for approval to Member States at the World Health Assembly in May 2019, and is expected to come into effect on January 1, 2022. The presentation of the new classification in 2019 will enable countries to plan for its implementation, prepare the necessary translations and train professionals accordingly [World Health Organization (WHO), 2018]. In texts published by WHO collaborators (Luciano, 2017), it has been suggested that the ICD-11 will include mood disorders within the mental and behavioral disorder category. However, until the final version is published, this information cannot be fully verified.

The two classifications (DSM-5 e IDC-10) offer different categorizations of depressive disorders, as shown in Table 1. The WHO includes depressive disorders in the mood disorders category, although this review only focuses on Sections F32, F33, F34, and F38, which include the most frequent depressive disorders and which, in turn, contain subsections that will be further specified later on.

**Table 1:** Depressive disorders according to the DSM-5 and the ICD-10.

**DSM-5**	**ICD-10**
Depressive disorders	Mood (affective) disorders F32, F33, F34, and F38
- Disruptive mood dysregulation disorder - Major depressive disorder - Persistent depressive disorder (dysthymia) - Premenstrual dysphoric disorder - Substance/medication-induced depressive disorder - Depressive disorder due to another medical condition - Other specified depressive disorder - Other unspecified depressive disorder	- Single episode (F32) - Recurrent depressive disorder (F33) - Persistent mood (affective) disorders (F34) - Other mood (affective) disorders (F38)

According to the DSM-5, depressive disorders all have one common feature, namely the presence of sad, empty or irritable mood, accompanied by somatic and cognitive changes that significantly affect the individual's capacity to function (DSM-5). They may become a serious health problem if allowed to persist for long periods of time and occur with a moderate-to-severe degree of intensity. One important consequence of depression is the risk of suicide, which is, according the World Health Organization (WHO) (2017), the second most common cause of death among young people aged between 15 and 29.

The main novelty offered by the DSM-5 in its section on depressive disorders is the introduction to *Disruptive mood dysregulation disorder* (which should not be diagnosed before the age of 6 or after the age of 18). This disorder is characterized by severe recurrent temper outbursts manifested verbally (e.g., verbal rages) and/or behaviorally (e.g., physical aggression toward people or property). These outbursts often occur as the result of frustration and in order to be considered a diagnostic criterion must be inconsistent with the individual's developmental level, occur three or more times per week for at least a year in a number of different settings (at home, at school, etc.) and be severe in at least one of these. This disorder was added to the DSM-5 due to doubts arising in relation to how to classify and treat children presenting with chronic persistent irritability as opposed to other related disorders, specifically pediatric bipolar disorder. The prevalence of this disorder has been estimated at between 2 and 5%, with male children and teenage boys being more likely to suffer from it than their female counterparts.

### Major Depressive Disorder

Major depressive disorder is characterized by a depressed mood most of the day, nearly every day, although in children and adolescents this mood may be irritable rather than depressed. The disorder causes a markedly diminished interest or pleasure in all, or almost all, activities most of the day, nearly every day, significant weight loss or gain, insomnia or hypersomnia, psychomotor agitation or retardation, fatigue or loss of energy, feelings of worthlessness, or excessive or inappropriate guilt, diminished ability to think or concentrate, recurrent thoughts of death, recurrent suicidal ideation without a specific plan, or a suicide attempt or a specific plan for committing suicide. These symptoms cause clinically significant distress or impairment in social, occupational, or other important areas of functioning. In the United States, the 12-month prevalence is ~7%, although it is three times higher among those aged between 18 and 29 than among those aged 60 or over. Moreover, the prevalence rates for women are ~1.5–3 times higher than for men.

### Persistent Depressive Disorder (Dysthymia)

Persistent depressive disorder (dysthymia) is a consolidation of DSM-5-defined chronic major depressive disorder and dysthymic disorder, and is characterized by a depressed mood for most of the day, for more days than not, for at least 2 years. In children and adolescents, mood can be irritable and duration must be at least 1 year. The DSM-5 specifies that patients presenting symptoms that comply with the diagnostic criteria for major depressive disorder for 2 years should also be diagnosed with persistent depressive disorder. When the individual in question is experiencing a depressive mood episode, they must also present at least two of the following symptoms: poor appetite or overeating, insomnia or hypersomnia, low energy or fatigue, low self-esteem, poor concentration, or difficulty making decisions and feelings of hopelessness. The prevalence of this disorder in the United States is 0.5%.

### Premenstrual Dysphoric Disorder

The diagnostic criterion for premenstrual dysphoric disorder states that, in the majority of menstrual cycles, at least five symptoms must be present during the last week before the start of menstruation, and individuals should start to feel better a few days later, with all symptoms disappearing completely or almost completely during the week after menstruation. The most important characteristics of this disorder are affective lability, intense irritability or anger, or increased interpersonal conflicts, markedly depressed mood and/or over-excitation, and symptoms of anxiety which may be accompanied by behavioral and somatic symptoms. Symptoms must be present during most menstrual cycles during the past year and must negatively affect occupational and social functioning. The most rigorous estimations of the prevalence of this disorder claim that 1.8% of women comply with the criterion but have no functional impairment, while 1.3% comply with the criterion and suffer functional impairment and other concomitant symptoms of another mental disorder.

### Substance/Medication-Induced Depressive Disorder

Substance/medication-induced depressive disorder is characterized by the presence of the symptoms of a depressive disorder, such as major depressive disorder, induced by the consumption, inhalation or injection of a substance, with said symptoms persisting after the physiological effects or the effects of intoxication or withdrawal have disappeared. Some medication may generate depressive symptoms, which is why it is important to determine whether the symptoms were actually induced by the taking of the drug or whether the depressive disorder simply appeared during the period in which the medication was being taken. The prevalence of this disorder in the United States is 0.26%.

### Depressive Disorder Due to Another Medical Condition

Depressive disorder due to another medical condition is characterized by the appearance of a depressed mood and a markedly diminished interest or pleasure in all activities within the context of another medical condition. The DSM-5 offers no information about the prevalence of this disorder.

The category *Other specified depressive disorder* is used when the symptoms characteristic of a depressive disorder appear and cause significant distress or impairment in social, occupational or other areas of functioning but do not comply with all the criteria of any depressive disorder, and the clinician opts to communicate the specific reason for this. In the *Other unspecified depressive disorder category*, on the other hand, the difference is that the clinician prefers not to specify the reason why the presentation fails to comply with all the criteria of a specific disorder and includes presentations about which there is insufficient information for giving a more specific diagnosis.

In the ICD-10, depressive disorders are included within the mood disorders category. The following disorders are analyzed below: single depressive episode, recurrent depressive disorder, and persistent mood (affective) disorders.

### Single Depressive Episode

The classification Single depressive episode distinguishes between depressive episodes of varying severity: mild, moderate, and severe without psychotic symptoms. Characteristics common to all of them include lowering of mood, reduction of energy, and decrease in daily activity. There is a loss of interest in formerly pleasurable pursuits, a decrease in the capacity for concentration, and an increase in tiredness, even during activities requiring minimum effort. Changes occur in appetite, sleep is disturbed, self-esteem and self-confidence drop, ideas of guilt or worthlessness are present and the symptoms vary little from day to day. In its mildest form, two or three of the symptoms described above may be present, and the patient is able to continue with most of their daily activities. When the episode is moderate, four or more of the symptoms are usually present and the patient is likely to have difficulty continuing with ordinary activities. In its most severe form, several of the symptoms are marked and distressing, typically loss of self-esteem and ideas of worthlessness or guilt. Suicidal thoughts and acts are common and a number of somatic symptoms are usually present. If the depressive episode is with psychotic symptoms, it is characterized by the presence of hallucinations, delusions, psychomotor retardation, or stupor so severe that ordinary social activities are impossible; there may be danger to life from suicide, dehydration, or starvation.

### Recurrent Depressive Disorder

Recurrent depressive disorder is characterized by repeated episodes of depression similar to those described above for single depressive episodes without mania. There may be brief episodes of mild mood elevation and over activity (hypomania) immediately after a depressive episode, sometimes precipitated by antidepressant treatment. The more severe forms of this disorder are very similar to manic-depressive depression, melancholia, vital depression, and endogenous depression. The first episode may occur at any age, from childhood to old age. The onset may be either acute or insidious and can last from a few weeks to many months. Recurrent depressive disorder can be mild or moderate, but in neither of these is there any history of mania. This section also includes recurrent depressive disorder currently in remission, in which the patient may have had two or more depressive episodes in the past, but has been free from depressive symptoms for several months.

### Persistent Mood [Affective] Disorders

Persistent mood [affective] disorders are persistent and usually fluctuating disorders in which the majority of episodes are not sufficiently severe to warrant being diagnosed as hypomanic or mild depressive episodes. Since they last for many years and affect the patient's normal life, they involve considerable distress and disability. This section also includes cyclothymia and dysthymia. Cyclothymia is a persistent instability of mood involving numerous periods of depression and mild elation, none of which are sufficiently prolonged to justify a diagnosis of bipolar affective disorder or recurrent depressive disorder. This disorder is frequently found among the relatives of patients with bipolar affective disorder and some patients with cyclothymia eventually develop bipolar affective disorder. For its part, dysthymia is a chronic depression of mood, lasting at least several years, which is not sufficiently severe, or in which individual episodes are not sufficiently prolonged, to justify a diagnosis of mild, moderate, or severe recurrent depressive disorder.

### Other Mood (Affective) Disorders

Finally, other mood (affective) disorders include any mood disorders that do not fall into the categories described above because they are not of sufficient severity or duration. They may be single, recurrent (brief), or specified episodes.

The manifestations and symptoms of depression vary in accordance with age and level of development. However, it is clear that the DSM-5 and the ICD-10 do not distinguish between adult and child depression, although by including disruptive mood dysregulation disorder, the DSM-5 does take into account the fact that children and young people aged between 7 and 18 may express their distress in other ways, through chronic, severe, and recurrent irritability manifested verbally and/or behaviorally. Similarly, major depressive disorder specifies that in children the mood may be irritable rather than depressed. However, no distinctions of this kind are found in the ICD-10, an absence which may lead to the faulty inference that the characteristics of child and adolescent depression are similar to those of adult depression.

## Explanatory Theories of Depression

Depressive disorders cannot be explained by any single theory, since many different variables are involved in their onset and persistence. The principal biological and psychological theories were therefore taken as the main references for this section. Subsequently, the contributions made by each of these theories regarding depression were studied by conducting searches in PubMed, Web of Science, Science direct, and Google Scholar. With the constant key words being depression, child depression and adolescent depression, the search for information cross-referenced a series of other key words also in accordance with the specific theory in question. Due to the importance of some seminal works in relation to the development of psychological theories of depression, certain authors have remained key references for decades. A total of 64 bibliographical references were used. The following is a summary of the various explanations for the onset of depression, according to the different theoretical frameworks.

### Biological Theories

If a mood disorder cannot be explained by family history or stressful life events, then it may be that the child or adolescent in question is suffering from a neurological disease. In such a case, depressive symptoms may manifest early in children and adolescents as epileptic syndromes, sleep disorders, chronic recurrent cephalalgias, several neurometabolic diseases, and intracranial tumors (Narbona, 2014).

#### Noradrenalin Deficit

Serotonin is a monoamine linked to adrenaline, norepinephrine, and dopamine which plays a key role, particularly in the brain, since it is involved in important life regulation functions (appetite, sleep, memory, learning, temperature regulation, and social behaviors, etc.), as well as many psychiatric pathologies (Nique et al., 2014). Serotonin modulates neuroplasticity, particularly during the early years of life, and dysfunctions in both systems contribute to the physiopathology of depression (Kraus et al., 2017). MRI tests in animals have revealed that a reduction in neuron density and size, as well as a reduction in hippocampal volume among depressive patients may be due to serotonergic neuroplasticity changes. Branchi (2011), however, argues that improving serotonin levels may increase the likelihood of both developing and recovering from the psychopathology, and underscores the role played by the social environment in this process. In this sense, Curley et al. (2011) point out that the quality of the social environment may influence the development and activity of neural systems, which in turn have an impact on behavioral, physiological, and emotional responses.

#### Endocrine Alterations

Age-related changes and the presence of biological risk factors, including endocrine, inflammatory or immune, cardiovascular and neuroanatomical factors, make people more vulnerable to depression (Clarke and Currie, 2009). Indeed, some studies suggest that depression may be linked to endocrine alterations: nocturnal cortisol secretions (Birmaher et al., 1996), nocturnal growth hormone secretion (Ryan et al., 1994), thyroid stimulating hormone secretion (Puig-Antich, 1987), melatonin and prolactin secretions (Waterman et al., 1994), high cortisol levels (Herane-Vives et al., 2018), or decreased growth hormone production (Dahl et al., 2000). Puberty and the accompanying hormonal and physical changes require special attention because it has been proposed that they could be associated with an increased incidence of depression (Reinecke and Simons, 2005).

#### Sleep Disorders

Sleep problems are often associated with situations of social deprivation, unemployment, or stressful life events (divorce, bad life habits, or poor working conditions) (Garbarino et al., 2016). It also seems, however, that sleep disorders are linked to the development of depression. This relationship occurs as a result of how insufficient sleep affects the hippocampus, heightening neural sensitivity to excitotoxic insult and vulnerability to neurotoxic challenges, resulting in a net decrease in gray matter in the hippocampus in the left orbitofrontal cortex (Novati et al., 2012).

For their part, Franzen and Buysse (2008) state that bidirectional associations between sleep disturbances (particularly insomnia) and depression make it more difficult to distinguish cause-effect relations between them. It is therefore unclear whether depression causes sleep disturbances or whether chronic sleep disturbances lead to the appearance of depression. What does seem clear, however, is that treating sleep disturbances (both insomnia and hypersomnia) may help reduce the severity of depression and accelerate recovery (Franzen and Buysse, 2008).

Longitudinal studies have identified insomnia as a risk factor for the onset or recurrence of depression in young people and adults (Sivertsen et al., 2014). In comparison with the non-clinical population, depressed children and adolescents report both trouble sleeping and longer sleep duration (Accardo et al., 2012).

For their part, Foley and Weinraub (2017) observed that, among preadolescent girls, early and later sleep problems directly or indirectly predicted a wide variety of social and emotional adjustment disorders (depressive symptoms, low school competence, poor emotion regulation, and risk-taking behaviors).

#### Altered Neurotransmission

Studies conducted over the past 20 years have shown that increased inflammation and hyperactivity of the hypothalamic–pituitary–adrenal (HPA) axis may explain major depression (Pariante, 2017). Some of the pathophysiological mechanisms of depression include altered neurotransmission, HPA axis abnormalities involved in chronic stress, inflammation, reduced neuroplasticity, and network dysfunction (Dean and Keshavan, 2017). Other studies report alterations in the brain structure: smaller hippocampus, amygdala, and frontal lobe (Whittle et al., 2014). Nevertheless, the underlying molecular and clinical mechanisms have yet to be discovered (Pariante, 2017). Major depressive disorder in children and adolescents has been associated with increased intracortical facilitation, a direct neurophysiological result of excessive glutamatergic neurotransmission. However, contrary to the findings in adults with depression, no deficits in cortical inhibition were found in children and adolescents with major depressive disorder (Croarkin et al., 2013).

#### Genetic Factors

Other studies have highlighted the importance of genetics in the onset of depression (40%) (Scourfield et al., 2003). It is important to recognize that a genetic predisposition to an excessive amygdala response to stress, or a hyperactive HPA axis (moderate hyperphenylalaninemia) due to stress during early childhood may trigger an excessive effect or alter an otherwise healthy psychological system (Dean and Keshavan, 2017). Kaufman et al. (2018) support a potential role for genes related to the homeobox 2 gene of Orthodenticle (OTX2) and to the OTX2-related gene in the physiopathology of stress-related depressive disorders in children. Furthermore, genetic anomalies in serotonergic transmission have been linked to depression. The serotonin-linked polymorphic region (5-HTTLPR) is a degenerate repeat in the gene which codes for the serotonin transporter (SLC6A4). The s/s genotype of this region is associated with a reduction serotonin expression, in turn linked to greater vulnerability to depression (Caspi et al., 2010).

For their part, Oken et al. (2015) claim that psychological disturbances may trigger changes in physiological parameters, such as DNA transcription, or may result in epigenetic modifications which alter the sensitivity of the neurotransmitter receptor.

### Psychological Theories

This section outlines the different psychological theories which have attempted to explain the phenomenon of depression. Depression is a highly complex disorder influenced by multiple factors, and it is clear that no single theory can fully explain its etiology and persistence. It is likely that a more eclectic outlook must be adopted if we are to make any progress in determining the origin, development, and maintenance of this pathology.

#### Attachment-Informed Theories

Attachment theory was the term used by Bowlby (1976) to refer to a specific conceptualization of human beings' propensity to establish strong and long-lasting affective ties with other people. Bowlby (1969, 1973) proposes that consistency, nurturance, protectiveness, and responsiveness in early interactions with caregivers contribute to the development of schemas or mental representations about the relationships of oneself with others, and that these schemas serve as models for later relationships. Bowlby's ethological model of attachment postulates that vulnerability to depression stems from early experiences which failed to satisfy the child's need for security, care and comfort, as well as from the current state of their intimate relations (Bowlby, 1969, 1973, 1988). Adverse early experiences can contribute to disturbances in early attachments, which may be associated with vulnerability for depression (Cummings and Cicchetti, 1990; Joiner and Coyne, 1999). Associations between insecure attachment among children and negative self-concept, sensitivity to loss, and an increased risk of depression in childhood and adolescence have been reported (Armsden et al., 1990; Koback et al., 1991; Kenny et al., 1993; Roelofs et al., 2006; Allen et al., 2007; Chorot et al., 2017). Relationships between secure attachment and depression seem also to be mediated by the development of maladaptive beliefs or schemas (Roberts et al., 1996; Reinecke and Rogers, 2001).

Thus, attachment theory has become a useful construct for conceptualizing many different disorders and provides valuable information for the treatment of depression (Reinecke and Simons, 2005).

Ainsworth described three attachment styles, in accordance with the child's response to the presence, absence, and return of the mother (or main caregiver): secure, anxious-avoidant, and anxious-resistant (Ainsworth et al., 1978). The least secure attachment styles may give rise to traumatic experiences during childhood, which in turn may result in the appearance of depressive symptoms.

Similarly, Hesse and Main (2000) argued that the central mechanism regulating infant emotional survival was proximity to attachment figures, i.e., those figures who help the child cope with frightening situations. Using Ainsworth's *strange situation* procedure, Main (1996) found that abused children engaged in more disorganized, disruptive, aggressive, and dissociative behaviors during both childhood and adolescence. Main (1996) also found that many people with clinical disorders have insecure attachment and that psychological-disoriented and disorganized children are more vulnerable.

For his part, Blatt (2004) explored the nature of depression and the life experiences which contribute to its appearance in more depth, identifying two types of depression which, despite a common set of symptoms, nevertheless have very different roots: (1) anaclitic depression, which arises from feelings of loneliness and abandonment; and (2) introjective depression, which stems from feelings of failure and worthlessness. This distinction is consistent with psychoanalytical formulations, since it considers defenselessness/dependency and desperation/negative feelings about oneself to be two key issues in depression.

Brazelton et al. (1975) found that at age 3 weeks, babies demonstrate a series of interactive behaviors during face-to-face mother-infant interactions. These behaviors were not found to be present in more disturbed interactions, which may trigger infant anxiety.

In a longitudinal study focusing on the relationship between risk of maternal depression and infant attachment behavior, Bigelow et al. (2018) analyzed babies at age 6 weeks, 4 and 12 months, finding that mothers at risk of depression soon after the birth of their child may have difficulty responding appropriately to their infant's attachment needs, giving rise to disorganized attachment, with all the psychological consequences that this may involve. Similarly, Beeghly et al. (2017) found that among infants aged between 2 and 18 months, greater maternal social support was linked to decreasing levels of maternal depressive symptoms over time, and that boys were more vulnerable than girls to early caregiving risks such as maternal depression, with negative consequences for mother-child attachment security during toddlerhood.

Authors such as Shedler and Westen (2004) have attempted to find solutions to the problems arising in relation to the DSM diagnostic categories, developing the Shedler Westen Assessment Procedure (SWAP-200) to capture the wealth and complexity of clinical personality descriptions and to identify possible diagnostic criteria which may better define personality disorders.

For their part, Ju and Lee (2018) argue that peer attachment reduces depression levels in at-risk children, and also highlight the curative aspect of attachment between adolescent peers.

#### Behavioral Models

The first explanations proposed by this model argued that depression occurs due to the lack of reinforcement of previously reinforced behaviors (Skinner, 1953; Ferster, 1966; Lewinsohn, 1975), an excess of avoidance behaviors and the lack of positive reinforcement (Ferster, 1966) or the loss of efficiency of positive reinforcements (Costello, 1972). A child with depression initially receives a lot of attention from his social environment (family, friends…), and behaviors such as crying, complaints or expressions of guilt are reinforced. When these depressive behaviors increase, the relationship with the child becomes aversive, and the people who used to accompany the child avoid being with him, which contributes to aggravating his depression (Lewinsohn, 1974). Low reinforcement rates can be explained by maternal rejection and lower parental support (Simons and Miller, 1987), by a lower rate of reinforcement offered to their children by mothers of depressed children (Cole and Rehm, 1986), or by low social competence (Shah and Morgan, 1996).

Depression is mainly a learned phenomenon, related to negative interactions between the individual and his or her environment (e.g., low rate of reinforcement or unsatisfactory social relations). These interactions are influenced by cognitions, behaviors and emotions (Antonuccio et al., 1989).

#### Cognitive Models

The attributional reformulation of the learned helplessness model (Abramson et al., 1978) and Beck's cognitive theory (Beck et al., 1979) are the two most widely-accepted cognitive theories among contemporary cognitive models of depression (Vázquez et al., 2000).

Learned helplessness is related to cognitive attributions, which can be specific/global, internal/external, and stable/unstable (Hiroto and Seligman, 1975; Abramson et al., 1978). Global attribution implies the conviction that the negative event is contextually consistent rather than specific to a particular circumstance. Internal attribution is related to the belief that the aversive situation occurs due to individual conditions rather than to external circumstances. Stable attribution is the belief that the aversive situation is unchanging over time (Miller and Seligman, 1975). People prone to depression attribute negative events to internal, stable and global factors and make external, unstable, and specific attributions for success (Abramson et al., 1978; Peterson et al., 1993), a cognitive style also present in children and adolescents with depression (Gladstone and Kaslow, 1995).

The Information Processing model (Beck, 1967; Beck et al., 1979) postulates that *depression is caused by particular stresses that evoke the activation of a schema that screens and codes the depressed individual's experience in a negative fashion* (Ingram, 1984, p. 443). Beck suggests that this distortion of reality is expressed in three areas, which he calls the “cognitive triad”: negative views about oneself, the world and the future as a result of their learning history (Beck et al., 1983). These beliefs are triggered by life events which hold special meaning for the subject (Beck and Alford, 2009).

#### Self-Control Model

This theory assumes that depression is due to deficits in the self-control process, which consists of three phases: self-monitoring, self-evaluation, and self-administration of consequences (Rehm, 1977; Rehm et al., 1979). In the self-monitoring phase, individuals attend only to negative events and tend to recognize only immediate, short-term consequences. In the self-evaluation phase, depressed individuals establish unrealistic evaluation criteria and inaccurately attribute their successes and failures. If self-evaluation is negative, in the self-administration of consequences phase the individual tends to engage very little in self-reinforcement and very frequently in self-punishment.

Both Rehm's self-control model (Rehm, 1977) and Bandura's conception of child depression (Bandura, 1977) assume that children internalize external control guidelines. These guidelines are related to family interaction patterns and both may contribute to the etiology or persistence of depression in children.

In a study conducted with children aged between 8 and 12 years, Kaslow et al. (1988) found that depressed children had a more depressive attributional style and more self-control problems.

#### Interpersonal Theory

This model, which is closely linked to attachment theories, aims to identify and find solutions for an individual's problems with depression in their interpersonal functioning. It suggests that the difficulties experienced are linked to unresolved grief, interpersonal disputes, transition roles and interpersonal deficits (Markowitz and Weissman, 1995).

Milrod et al. (2014) argue that pathological attachment during early childhood has serious consequences for adults' ability to experience and internalize positive relationships.

Similarly, various different studies have highlighted the fact that one of the variables that best predicts depression in children is peer relations (Bernaras et al., 2013; Garaigordobil et al., 2017).

#### Stressful Life Events

Studies focusing on the adult population have reported that between 60 and 70% of depressed adults experienced one or more stressful events during the year prior to the onset of major depression (Frank et al., 1994). In children and adolescents, modest associations have been found between stressful life events and depression (Williamson et al., 1995). For their part, Shapero et al. (2013) found that people who had suffered severe emotional abuse during childhood experienced higher levels of depressive symptoms when faced with current stressors. Sokratous et al. (2013) argue that the onset of depression is not only triggered by major stressful events, but rather, minor life events (dropping out of school, your father losing his job, financial difficulties in the family, losing friends, or the illness of a family member) may also influence the appearance of depressive symptoms.

Events such as the loss of loved ones, divorce of parents, mourning or exposure to suicide (either individually or collectively) have all been associated with the onset of depression in childhood (Reinherz et al., 1993). Factors such as a history of additional interpersonal losses, added stress factors, a history of psychiatric problems in the family and prior psychopathology (including depression) increase the risk of depression in adolescents (Brent et al., 1993). Birmaher et al. (1996) found that prior research into stressful life events in relation to early-onset depression had been based on data obtained from self-reports, making it difficult to determine the causal relationship, since events may be both the cause and consequence of depression.

However, not everyone exposed to this kind of traumatic experience becomes depressed. Personality and the moment at which events occur are both involved in the relationship between depression and stressful life events, although biological factors such as serotonergic functioning (Caspi et al., 2010) also exert an influence.

#### Sociocultural Models

These models postulate that cultural variables are responsible for the appearance of depressive symptoms. These variables are mainly acculturation and enculturation. In acculturation, structural changes are observed (economic, political, and demographic), along with changes in people's psychological behavior (Casullo, 2001). Some studies link increased suicide rates with economic recession (Chang et al., 2013; Reeves et al., 2014). Enculturation occurs when the older generation invites, induces or forces the younger generation to adopt traditional mindsets and behaviors.

In an attempt to better understand the influence of culture and family on depressive symptoms, Lorenzo-Blanco et al. (2012) tested an acculturation, cultural values and family functioning model with Hispanic students born in the United States. The results revealed that both family conflict and family cohesion were related to depressive symptoms.

Another study carried out with girls aged 7–10 years (Evans et al., 2013) observed that internalizing an unrealistically thin ideal body predicted disordered eating attitudes through body dissatisfaction, dietary restraint and depression.

Finally, the importance of family interactions in the onset of depressive symptoms cannot be overlooked. Parenting style has been identified as a key factor in children's and adolescents' psychosocial adjustment (Lengua and Kovacs, 2005). Parental behavior has been studied from two different perspectives: warmth and control. Warmth is linked to aspects such as engagement and expression of affection, respect, and positive concern by parents and/or principal caregivers (Rohner and Khaleque, 2003). In this sense, prior studies have identified a significant association between parental warmth and positive adjustment among adolescents (Barber et al., 2005; Heider et al., 2006). Rohner and Khaleque (2003) argue that children's psychological adjustment is closely linked to their perception of being accepted or rejected by their principal caregivers, and other studies have found that weaker support from parents is associated with higher levels of depression and anxiety among adolescents (Yap et al., 2014).

Similarly, Jaureguizar et al. (2018) found that a low level of perceived parental warmth was linked to high levels of clinical and school maladjustment, and that the weaker the parental control, the greater the clinical maladjustment. These authors also found that young people with negligent mothers and authoritarian fathers had higher levels of clinical maladjustment.

In short, according to the different theories, depression may be due to (1) biological reasons; (2) insecure attachment; (3) lack of reinforcement of previously-reinforced behaviors; (4) negative interpersonal relations and relations with one's environment and the resulting negative consequences; (5) attributions made by individuals about themselves, the world and their future; and (6) sociocultural changes. It is likely that no single theory can fully explain the genesis and persistence of depression, although currently, negative interpersonal relations and relations with one's environment and sociocultural changes (economic, political, and demographic) may explain the observed increase in the prevalence of depression.

## Evaluation Instruments

Many different evaluation instruments can be used to measure child and adolescent depression. Tables 2, 3 outline the ones most commonly used in scientific literature. Table 2 summarizes the main self-administered tests that specifically measure child and adolescent depression, while Table 3 presents tests that measure child and adolescent depression among other aspects (i.e., broader or more general tests). Finally, Table 4 summarizes the main hetero-administered psychometric tests for assessing this pathology.

**Table 2:** Self-administered psychometric tests designed specifically for evaluating child and adolescent depression.

**Name**	**References**	**Num. items**	**Dimensions (variables measured)**	**Target age group**	**Psychometric characteristics**	**Spanish adaptation**
Children's Depression Scale (CDS)	Lang and Tisher, 1978	66	Depressive total: Affective Response, Social Problems, Self-esteem, Pre-occupation with own Sickness or Death, Guilt and Various Depressives. Positive Total: Mood-Joy and Various Positives.	8–16	K-R20 = 0.91; Alpha = 0.92–0.94; Guttman split-half coefficient = 0.90; Test-retest reliability = 0.74 (Bath and Middleton, 1985; Tisher et al., 1992)	Seisdedos, 2003
Children's Depression Inventory (CDI)	Kovacs, 1985	27	Scales: emotional problems, functional problems. Subscales: negative mood/physical symptoms, negative self-esteem, interpersonal problems, ineffectiveness	7–17	Alpha = 0.75–0.90; κ = 0.76; K-R20 = 0.80–0.94; Test-retest reliability = 0.62–0.82; Sensitivity = 0.81–0.95; Specificity = 0.64–0.96; PPV = 0.21–0.90; NPPV = 0.63–1.00 (view review by Stockings et al., 2015)	Del Barrio and Carrasco, 2004
Center for Epidemiological Studies Depression Scale for Children (CES-DC)	Weissman et al., 1980	20	Total depression	12–18	Alpha = 0.86–0.89; Sensitivity = 0.71–0.82; Specificity = 0.62–0.90; PPV = 015; NPV = 0.96 (view review by Stockings et al., 2015)	Soler et al., 1997
Depression Self-Rating Scale for Children (DSRS)	Birleson, 1981	18	Total depression	8–14	Alpha = 0.86 Test-retest reliability = 0.80; Sensitivity = 0.67; Specificity = 0.77; PPV = 0.15; NPV = 0.97 (Birleson et al., 1987)	No
Reynolds Adolescent Depression Scale (RADS)	Reynolds, 1987	30	Total depression, dysphoric mood, Anhedonia/negative affect, Negative self-evaluation, somatic complaints	13–17	Alpha = 0.92 (view review by Stockings et al., 2015); Sensitivity = 0.86; Specificity = 0.49; PPV = 0.69; NPV = 0.72 (Krefetz et al., 2002)	Del Barrio et al., 1996
Reynolds Child Depression Scale (RCDS)	Reynolds, 1989	30	Total depression	7–13	Alpha = 0.85–0.91 Test-retest reliability = 0.82–0.85 (Reynolds, 1989); Sensitivity = 0.79; Specificity = 0.87 (Figueras et al., 2008)	Figueras et al., 2008
Mood and Feelings Questionnaire (MFQ), and Short Mood and Feelings Questionnaire (SMFQ)	Angold et al., 1995	32 (MFQ) 13 (SMFQ)	Total depression	8–18	MFQ: Alpha = 0.90–0.93; AUC = 0.86 (95% CI: 0.81, 0.91), Sensitivity = 0.84; Specificity = 0.70. SMFQ: Alpha = 0.87–0.89; AUC = 0.86 (95% CI: 0.80, 0.91); Sensitivity = 0.84; Specificity = 0.68 (Thabrew et al., 2017)	No
Beck Depression Inventory (BDI-II)	(Beck et al., 1996)	21	Total depression	13 and over	Alpha = 0.92–0.94; Sensitivity = 0.74–0.88; Specificity = 0.70–0.92; PPV = 0.76–0.85; NPV = 0.67–0.95 (view review by Stockings et al., 2015)	Sanz and Vázquez, 1998
Revised Child Anxiety and Depression Scale—RCADS	Chorpita et al., 2000	Two versions: with 47 and 30 items	Anxiety and separation disorders. Social phobia, Generalized anxiety, Panic, Obsessive compulsive disorder and Major depressive disorder. Total Anxiety and Depression Score	8-18	Alpha = 0.78; sensitivity = 0.74; specificity = 0.77 for the Major Depression scale (MDD). RCADS MDD scale correlated positively and significantly with the CDI	RCADS: (Sandín et al., 2009); RCADS-30: (Sandín et al., 2010)
Kutcher Adolescent Depression Scale (KADS)	Leblanc et al., 2002	16 (long), 11 (short), and 6 (brief)	Total depression	6–18	KADS-16 Alpha = 0.82; AUC = 0.85 KADS-11: Alpha = 0.84; AUC = 0.94, (95%CI: 0.91, 0.97); Sensitivity = 0.89; Specificity = 0.90 KADS-6: Alpha = 0.80 Sensitivity = 0.92 Specificity = 0.71; PPV = 0.10 for total sample and.26 for clinical sample; NPV = 1.0 for the total sample and.99 for the clinical sample (Leblanc et al., 2002; Brooks et al., 2003; Zhou et al., 2016)	There is a Spanish version, but no data are available regarding its validation.
Cuestionario Educativo Clínico de ansiedad y depresión (*Clinical Educational Anxiety and Depression Questionnaire*) (CECAD)	Lozano et al., 2007	50	Depression, anxiety, worthlessness, irritability, problems with thinking and psycho-physiological symptoms	7-adulthood	Alpha = > 0.83 Omega coefficient = 0.77–0.87. Correlations with CDI between 0.26 and 0.76.	–

*KR-20, Kuder-Richardson coefficient (formula 20); κ, Cohen's kappa reliability co-efficient; PPV, Positive predictive value; NPV, Negative predictive value; AUC, Area under the Receiver Operating Characteristic Curve (AUC)*.

**Table 3:** Self-administered general psychometric tests which, among other variables, also assess child and adolescent depression.

**Name**	**References**	**Num. items**	**Dimensions (variables measured)**	**Target age group**	**Psychometric characteristics**	**Spanish adaptation**
Symptom Checklist-90. SCL-90	Derogatis and Cleary, 1977	90	Somatization, obsessive-compulsive, interpersonal sensitivity, depression, anxiety, hostility, phobic anxiety, paranoid ideation, psychoticism. Global severity index, Positive Symptom Distress Index, Positive Symptom Total.	13 and over	Alpha = 0.98	González De Rivera et al., 2002
Pediatric Symptom Checklist (PSC)	Jellinek et al., 1979	35	Attention, internalizing symptoms, and externalizing symptoms	3-16	Alpha = .91	Version for adolescents: (Lemos et al., 1992)
Child Behavior Checklist (CBCL)	Achenbach and Edelbrock, 1985	133	Scales: Withdrawal, somatic complaints, anxiety/depression, social problems, thought problems, attention problems, rule-breaking behavior and aggressive behavior	4–18	Alpha = between 0.72 and 0.97	Rubio-Stipec et al., 1990
Behavior Assessment System for Children (BASC-2) Sistema de Evaluación de la Conducta de Niños y Adolescentes	Reynolds and Kamphaus, 1992	Total: 146. 15 items on depression	Negative attitude to school, negative attitude to teachers, atypicality, external locus of control, social stress, anxiety, depression, sense of inadequacy, interpersonal relations, relations with parents, self-esteem, self-reliance, clinical maladjustment, school maladjustment, personal adjustment, emotional symptoms index	8–12	Alpha = between 0.70 and 0.80	González et al., 2004
Self-administered Psychiatric Scales for Children and Adolescents (SAFA)	Cianchetti and Sannio Fascello, 2001	174	Anxiety, depression, obsessive-compulsive symptoms, eating disorders, hypochondria, somatic symptoms and phobias	8–18	Alpha = 0.80	No
Beck Youth Inventories (BYI-2)	Beck et al., 2005	100	Depression, anxiety, anger, disruptive behavior and self-concept.	7–18	Alpha = between 0.90 and 0.95	No (scheduled for the near future)

**Table 4:** Hetero-administered psychometric tests for assessing child and adolescent depression.

**Name**	**References**	**Num. items**	**Dimensions (variables measured)**	**Administered by**	**Target age group**	**Psychometric characteristics**	**Spanish adaptation**
Children's Depression Rating Scale-Revised (CDRS-R)	Poznanski et al., 1979	17	Total depression	Clinical personnel (interviews with child and parents)	6–12	Alpha = .85	No
Escala para la evaluación de la depresión para maestros (*Teachers' scale for evaluating depression*) (ESDM)	Domènech-Llaberia and Polaino-Lorente, 1990	16	Performance, social interaction, inhibited depression, and anxious depression	Teachers	8–12	Alpha = 0.88	–
Diagnostic Interview for Children and Adolescents–Revised (DICA-R)	Reich et al., 1991	1–2 h	Disruptive behavior disorders, mood disorders, anxiety disorders, eating disorders, and elimination disorders	Clinical personnel (interviews with child and parents)	8–18	High intra-rater reliability and moderate agreement between parents-children/adolescents (see Ezpeleta et al., 1995).	Ezpeleta et al., 1995
Semistructured Clinical Interview for Children and Adolescents (SCICA)	McConaughy and Achenbach, 1994	224	Anxiety, depression, motor/language problems, attention problems, self-control problems, aggression, somatic complaints	Clinical personnel	6–18	Mean test-retest reliability: 0.78	No
The Kiddie Schedule for Affective Disorders and Schizophrenia-Present and Lifetime (K-SADS-PL)	Kaufman et al., 1996	82+5 modules	Major depression, dysthymia, mania, hypomania, cyclothymia, bipolar disorder, schizoaffective disorder, schizophrenia, schizophreniform disorder, brief reactive psychosis, panic disorder, agoraphobia, separation anxiety disorder, avoidant disorder of childhood and adolescence, simple phobia, social phobia, generalized anxiety, obsessive compulsive disorder, attention deficit hyperactivity disorder, conduct disorder, oppositional defiant disorder, enuresis, encopresis, anorexia nervosa, bulimia, transient tic disorder, Tourette's disorder, chronic motor or vocal tic disorder, alcohol abuse, substance abuse, post-traumatic stress disorder, and adjustment disorders	Clinical personnel	6–18	High convergent validity and limited divergent validity (Lauth et al., 2010).	No
Diagnostic Interview Schedule for Children (DISC)	Shaffer et al., 2000	1–2 h	Anxiety disorders, mood disorders, disruptive disorders, substance abuse, others (anorexia, bulimia, enuresis/encopresis, selective mutism, schizophrenia, etc.)	Clinical personnel (interviews with child and parents)	6–17	No validity data available. Acceptable test-retest reliability (Shaffer et al., 2000)	Bravo et al., 2001
Screening de problemas emocionales y de conducta infantil (*Screening for emotional and behavioral problems in children*) (SPECI)	Garaigordobil and Maganto, 2012	10 min	Withdrawal, somatization, anxiety, infantile-dependent, problems with thinking, attention-hyperactivity, disruptive behavior, academic performance, depression, and violent behavior	Teachers	5–12	Alpha = 0.82	–

As shown in the tables above, there are several self-administered instruments that can be used with children from age 6 to 7 onwards, although their duration should be taken into consideration in order to avoid overtiring subjects. While it is clear that an effort has been made to design shorter measures (compare, for example, the 66 items of the CDS with the 16 items of the longest version of the KADS), the duration of the test should not be the only aspect taken into account when selecting an evaluation instrument.

One of the most widely used instruments to measure child depression in the scientific literature is the Children's Depression Inventory-CDI (Kovacs, 1985), which is based on the Beck Depression Inventory-BDI (Beck and Beamesderfer, 1974). Thus, it is based on Beck's cognitive theory of depression. Following this same theoretical line, the Children's Depression Scale-CDS (Lang and Tisher, 1978) was designed, but in this case, this instrument was not created based on another instrument previously designed for adult population (as in the case of the CDI), but instead from its beginnings, it was conceived exclusively to assess child depression. Chorpita et al. (2005) explain that the CDI measures a broader construct of negative affectivity rather than depression as a separate construct, and that it may be useful for screening for trait dimensions or personality features, whereas other instruments, such as the Revised Child Anxiety and Depression Scale-RCADS (Chorpita et al., 2000), measure a specific clinical syndrome.

Table 2 describes many other instruments that are very useful as screening tests for depression and depressive disorder, such as the Center for Epidemiological Studies Depression Scale for Children-CES-DC (Weissman et al., 1980) (based on the Center for Epidemiological Studies Depression Scale for Adults, CES-D; Radloff, 1977), the Mood and Feelings Questionnaire-MFQ (Angold et al., 1995), or the Depression Self-Rating Scale for Children-DSRS (Birleson, 1981). This last one, for example, is useful to measure moderate to severe depression in childhood and is based on the operational definition of depressive disorder, that is, a specific affective-behavior pattern that implies an impairment of a child's or adolescent's ability to function effectively in his/her environment (Birleson, 1981).

The cognitive and affective component of depression is the one that is most present in the instruments described in Table 2. In fact, for example, the Short Mood and Feelings Questionnaire (SMFQ) includes the cognitive and affective items from the original MFQ item pool, in addition to some items related to tiredness, restlessness, and poor concentration (Angold et al., 1995). In the SMFQ, more than half of the items from the MFQ were removed, and even so, high correlations between the MFQ and the SMFQ were found (Angold and Costello, 1995), which may be indicating that the really important items were the cognitive and affective items that were maintained. Reynolds et al. (1985) defended that children could accurately report their cognitive and affective characteristics, so “*if one wishes to know how a child feels, ask the child”* (Reynolds et al., 1985, p. 524).

Depending on the specific aim of the evaluation or research study, a broader diagnostic measure, such as those outlined in Table 3, may also provide valuable information. Finally, it is worth noting that only two hetero-administered instruments were found for teachers, with all others being clearly oriented toward the clinical field. In this sense, special emphasis should be placed on the need to develop valid and reliable instruments for teachers, since they may be key agents for detecting symptoms among their students. While it is important to train teachers in this sense, it is also important to provide them with instruments to help them assess their students. The instruments that are currently available have produced very different results as regards their correlation with students' self-reported symptoms, although in general, teachers tend to underestimate their students' depressive symptoms (Jaureguizar et al., 2017).

## Child and Adolescent Depression Prevention Programs in the School Environment

Extant scientific literature was reviewed in order to summarize the main depression prevention programs for children and adolescents in school settings. The databases used for conducting the searches were PubMed, PsycINFO, Web of Science, Scopus, Science Direct, and Google Scholar, along with a range of different manuscripts. With the constant key word being depression, the search for information cross-referenced a series of other key words also, namely: “child^*^ OR adolescent^*^,” “prevent^*^program,” and “school OR school-based.” Searches were conducted for information published between January 1, 1970 and December 31, 2017.

First, articles were screened (i.e., their titles and abstracts were read and a decision was made regarding their possible interest for the review study). The inclusion criteria were that the study analyzed all the research subjects of the review study (depression, childhood, or adolescence and prevention programs in school settings), that study participants were aged between 6 and 18, that the study was published in a peer-reviewed journal and that it was written in either English or Spanish. Review studies and their references were also analyzed. Studies focusing mainly on psychiatric disorders other than depression were excluded.

Finally, 39 studies were selected for the review, which explored 8 prevention programs that are outlined in Table 5. In general terms, child depression prevention programs are divided into two main categories: universal programs for the general population, and targeted programs aimed at either the at-risk population or those with a clear diagnosis. Although scientific literature reports that targeted programs obtain better outcomes than universal ones, the latter type nevertheless offer certain advantages, since they reach a larger number of people without the social stigma attached to having been specially selected (Roberts et al., 2003; Huggins et al., 2008). Thus, the ideal context for instigating universal child depression prevention programs is the school environment.

**Table 5:** School-based child and adolescent depression prevention programs.

**Name**	**References**	**Target age group**	**Aims**	**Type**	**Characteristics**	**Results**
Penn Resiliency Program (PRP)	Gillham et al., 1990	8–15	To raise awareness of the relationship between cognition, emotion and behavior, help youngsters develop social and decision-making skills and foster optimism	U	- Cognitive-behavioral perspective - 12 sessions, each lasting 90–120 min - Contents: cognitive restructuring, maladaptive coping strategies, attributional styles, assertiveness, negotiation, relaxation, decision-making, social skills	Significant reduction in depression levels assessed using the CDI (Gillham, 1994; Gillham et al., 1995; Quayle et al., 2001; Chaplin et al., 2006; Cardemil et al., 2007) as well as in anxiety levels and behavioral problems, and fostering of wellbeing and optimism. Other universal (Pattison and Lynd-Stevenson, 2001; Gillham et al., 2007) and targeted studies (Gillham and Reivich, 1999; Roberts et al., 2003a, 2004) failed to find any evidence that the program had any impact on the variables analyzed, in either the short or long term. The targeted study by Gillham et al. (2006) found that PRP did not significantly prevent depressive disorders but significantly prevented depression, anxiety, and adjustment disorders (when combined) among high-symptom participants
Coping with Stress Course (CWSC)	Clarke et al., 1995	13–17	To challenge irrational thoughts, cope with negative moods, overcome passivity, and reach agreements with parents and peers; social skills training	T	- Target population: Adolescents with some known increased risk of depression: past episode of depression; persistent sub-diagnostic dysphoria and/or other depressive symptoms; depressed parents; pregnant, single teen mother; other known risk factors for depression - Based on Beck's (Beck et al., 1979) and Ellis' (Ellis and Harper, 1961) cognitive therapy - 15 sessions lasting 45 min and 8 sessions lasting 90 min - Contents: depression and its relationship with stress, training in cognitive restructuring skills and the modification of irrational thoughts	Significant reduction in depression levels and the risk of reappearance at posttest and during follow-up (8, 12, and 18 months) (Clarke et al., 1995, 2001; Garber et al., 2009) (depressive symptoms assessed by the Center for Epidemiologic Studies-Depression Scale-CES-D). Participants who were currently on antidepressant medication were excluded from these studies
Aussie optimism program	Rooney et al., 2000	6–11	To intervene in risk and protection factors for depression and anxiety (cognitive, emotional, and social characteristics).	U	- Cognitive-behavioral perspective - 10 sessions, each lasting 60 min - Contents: identifying negative beliefs about oneself and one's present and future circumstances, identifying and regulating emotions, engaging in pleasurable activities, working with hierarchies of situations which generate fear and learning to relax (Rooney et al., 2013)	- Rooney et al. (2006) applied the program to 72 children aged between 8 and 9 and found both a significant reduction in depression levels (assessed using the CDI) and more positive causal attributions at posttest and during short-term follow up, although these results were not found to persist in the long term. The effect sizes observed were low (η^2^ = 0.09 for the symptoms of depression and η^2^ = 0.16 for attributional style). - The program was also found to reduce depressive symptoms (assessed using the CDI) in a study with 47 female 7th grade students, with a 6-month follow up (Quayle et al., 2001) Resourceful Adolescent Program-Adolescents (RAP-A)
Resourceful Adolescent Program- Adolescents (RAP-A)	Shochet et al., 2001	12–15	To identify and challenge irrational thoughts, provide training in social skills and problem solving and help prevent conflicts with parents and peers	U	- Cognitive-behavioral perspective - 11 sessions, each lasting 40–50 min - Contents: cognitive restructuring, problem-solving, social skills, and communication training	Significant results in preventing depression in random groups at posttest (measured using the BDI-II and RADS) but not during follow up, at least according to the BDI-II (the effect persisted according to the RADS) (Merry et al., 2004), although when non-random groups were used the results were also significant during follow up (Shochet et al., 2001)
FRIENDS	Barrett and Turner, 2001	7–16	To reduce the incidence of anxiety and depression, emotional distress and social problems, teaching children how to cope with anxiety, both now and in the future	U	- Cognitive-behavioral perspective - 10 sessions, each lasting 120 min + 2 booster sessions - Contents: one's own and other people's emotions, relaxation, trying to do your best, planning steps, making time to have fun together, friendship and family skills, being happy, etc.	Reduced anxiety levels, although the results regarding reduced depression levels are more limited (Barrett and Turner, 2001; Lowry-Webster et al., 2001; Lock and Barrett, 2003). In Barrett and Turner's study with 489 children aged between 10 and 12, the effect size for depression (measured using the CDI) at posttest was 0.09. Curiously enough, self-reported depression decreased slightly in the psychologist-led condition, while slight increases were noted in the teacher-led condition, although these increases were statistically, but not clinically, significant (Barrett and Turner, 2001)
Problem Solving for Life (PSFL)	Spence et al., 2003	13–15	Cognitive restructuring, problem solving	U	- Cognitive-behavioral perspective - 8 sessions, each lasting 45–50 min - Contents: challenging maladaptive thoughts, coping with problems	No significant results were found as regards preventing depressive symptoms (Spence et al., 2005; Sheffield et al., 2006), although an improvement was observed in all the variables studied in all study groups (those that did and those that did not participate in the program) (Sheffield et al., 2006)
Interpersonal Psychotherapy-Adolescent Skills Training (IPT-AST)	Young and Mufson, 2003	11–16	Training in social skills, coping with life transitions, and overcoming interpersonal deficits	T	- Target population: adolescents with elevated symptoms of depression - Cognitive-behavioral perspective - 10 sessions, each lasting 90 min - Contents: social and communication skills	Immediate reduction in depressive symptoms, although the benefits did not persist longer than 6 months (Horowitz et al., 2007; Young et al., 2010) (depressive symptoms assessed using the Center for Epidemiologic Studies-Depression Scale-CSD-D and the Children's Depression Rating Scale (CDRS)
Adolescents Coping with Emotions (ACE)	Sheffield et al., 2006	14–15	To prevent or reduce depression levels, improving coping skills, and fostering resilience	T	- Target population: adolescents with elevated symptoms of depression (those scoring in the top 20% on the combined scores, sum of standardized scores, the Children's Depression Inventory (CDI) and the Center for Epidemiologic Studies—Depression Scale (CES-D). - Cognitive-behavioral perspective - Applied by teachers - 8 sessions, each lasting 60 min - Contents: cognitive restructuring, training in social skills and assertiveness, negotiation and problem-solving skills, recognition of one's own achievements	Significant reduction in depressive symptoms and negative thoughts in girls after 6 months (Kowalenko et al., 2005), but not during the 12-month follow up (Sheffield et al., 2006). Depressive symptoms were assessed using the CDI and CES-D
FORTIUS	Méndez et al., 2013	8–13	To psychologically strengthen participants at a cognitive, emotional and behavioral level	U	−12 sessions lasting 50–60 min +2 booster sessions one month later +1 final session 3 months later - Contents: understanding and controlling negative emotions, social skills, and the ability to organize everyday activities, detection and restructuring of negative thoughts, problem solving, decision making, and positive self-instruction	No significant differences were found in depression (measured using the CDI) at posttest, although a reduction was observed during follow up (12 months) in girls (Orenes, 2015)

*Type: T, targeted; U, universal*.

Table 5 outlines the most important child depression prevention programs carried out in the school context. They are all cognitive-behavioral programs implemented either by psychologists or teachers with specialist training, consisting of between 8 and 15 sessions. Only a few universal programs designed to prevent the symptoms of depression focus on younger children, since most are targeted mainly at the adolescent population (Gillham et al., 1995; Barrett and Turner, 2001; Farrell and Barrett, 2007; Essau et al., 2012; Gallegos et al., 2013; Rooney et al., 2013). Indeed, in the present review, only four universal child depression prevention programs were found that were aimed at a younger age group (between 8 and 12): the Penn Resiliency Program, FRIENDS, the Aussie Optimism Program, and FORTIUS (see Table 5).

As shown in the table, the results of the various programs outlined are not particularly positive, since on many occasions the effects (if there are any) are not sustained over time or are limited in scope (being dependent on who applies the program or on the sex of the participant, etc.). Nor is the distinction between universal and targeted programs particularly clear as regards their effects, since although targeted programs may initially appear to be more effective, their impact is not found to be sustained in the long term.

Greenberg et al. (2001) argue that researchers should explain whether their prevention programs focus on one or various microsystems (basically family and school), mesosystems or exosystems, etc. (following the model described by Bronfenbrenner, 1979), or are centered exclusively on the individual and his or her environment, since this will influence the results reported. These same authors conclude that programs focused exclusively on children and adolescents themselves are less effective than those which aim to “educate” subjects and bring about positive changes in their family and school environments.

As Calear and Christensen (2010) point out in their review, some authors suggest that the fact that some targeted programs are aimed at people with high levels of depressive symptoms entails a broader range of possibilities for change; however, this does not help us understand why these changes are not sustained over time. Thus, further research is required in this field in order to identify what specific components of those programs observed to be effective actually have a positive impact on the level of depressive symptoms, how these programs are developed, who implements them and whether or not their effects are sustained in the short, medium, and long term.

## Clinical Treatments for Depression

In order to draft this section, a search was conducted for the most commonly-used therapies with proven efficacy for treating depression. The databases used were PubMed, Web of Science, Science direct, and Google Scholar. The key words used in the search were treatment, depression, child depression, and adolescent depression. A total of 30 bibliographic references were used in the drafting of this summary, including the major contribution made by The American Psychological Association's Society of Clinical Psychology (American Psychological Association, Society of Clinical Psychology (APA), 2017) regarding the most effective psychological methods for treating depression.

Although the World Health Organization (WHO) (2017) claims that prevention programs reduce the risk of suffering from depression, it has yet to be ascertained what type of programs and what contents are the most effective. The WHO also states that there are effective treatments for moderate and severe depression, such as psychological treatments (behavioral activation, cognitive behavioral therapy, and interpersonal psychotherapy) and antidepressant drugs (although it also warns of adverse effects), as well as psychosocial treatments for cases of mild depression. Moreover, a study conducted with adolescents by Foster and Mohler-Kuo (2018) found that the combination of cognitive-behavioral therapy and fluoxetine (antidepressant drug) was more effective than drug therapy alone.

The efficacy of treatment with antidepressants has been called into question for some years now. Iruela et al. (2009) claim that tricyclic antidepressants (imipramine, clomipramine, amitriptyline) are not recommended in childhood and adolescence since no benefits other than the placebo effect have been proven and furthermore, they generate major side effects due to their cardiotoxicity. They are therefore particularly dangerous in cases of attempted suicide. These same authors also advise against the use of monoamine oxidase inhibitors (MAOIs) due to dietary restrictions, interactions with other medication and the lack of clinical trials with sufficiently large groups which guarantee their efficacy. SSRIs or serotonergic antidepressants are the ones that have been most extensively studied in this population. The most effective is fluoxetine, the use of which is recommended in association with cognitive psychotherapy for cases of moderate and severe child depression.

On another hand, Wagner and Ambrosini (2001) analyzed the efficacy of pharmacological treatment in children and adolescents and stated that, at best, antidepressant therapy for depressed youth was moderately effective. Peiró et al. (2005) indicate that there is a great debate about the safety of selective serotonin reuptake inhibitors (SSRIs) in childhood. SSRIs, except for fluoxetine in the United States, have never been authorized by any agency for use in children or adolescents, mainly because of the risk of suicide to which they are associated. In 1991, the Food and Drugs Administration (FDA) claimed that there was insufficient evidence to confirm a causal association between SSRIs and suicide. Vitiello and Ordoñez (2016) conducted a systematic review of the topic and found more than 30 controlled clinical trials in adolescents and a few studies with children. Most studies found no differences between studies that administered drugs and those that used placebo, but they did find fluoxetine to be effective. They noted that antidepressants increased the risk of suicide (suicidal ideation and behaviors) compared to studies that had used placebos. The authors recommend using antidepressants with caution in young people and limiting them to patients with moderate to severe depression, especially when psychosocial interventions are not effective or are not feasible.

As regards the effectiveness of psychodynamic treatments, Luyten and Blatt (2012) advocate the inclusion of psychoanalytic therapy in the treatment of child, adolescent and adult depression. After conducting a review of both the theoretical assumptions of psychodynamic treatments of depression and the evidence supporting the efficacy of these interventions, these authors concluded that brief psychoanalytic therapy (BPT) is as effective in treating depression as other active psychotherapeutic treatments or pharmacotherapy, and its effects tend to be maintained in the longer term. They also observed that the combination of BPT and medication obtained better results than medication alone. Longer-term psychoanalytic treatment (LTPT) was found to be effective for patients suffering from chronic depression and co-morbid personality problems. Together, the authors argue, these findings justify the inclusion of psychoanalytic therapy as a first-line treatment in adult, child, and adolescent depression.

In a qualitative study carried out by Brown (2018) on parents' expectations regarding the recovery of their depressed children, a direct relationship was observed between said expectations and type of attachment. Parents who remained more passive and expected expert helpers to fix their child experienced reduced hope months after finishing the program. However, when parents changed their interactions with their child and adopted more positive expectations regarding their cure, they felt a more sustained sense of hope. Moreover, when parents themselves participated in therapy sessions, as part of their child's treatment, they felt greater hope and effectiveness in contributing to their child's recovery.

The American Psychological Association's Society of Clinical Psychology [American Psychological Association, Society of Clinical Psychology (APA), 2017] has published a list of psychological treatments that have been tested with the most scientific rigor and which, moreover, have been found to be most effective in treating depression. These treatments are as follows:

– Self-Management/Self-Control Therapy (Kanfer, 1970). Depression is due to selective attention to negative events and immediate consequences of events, inaccurate attributions of responsibility for events, insufficient self-reinforcement, and excessive self-punishment. During therapy, the patient is provided with information about depression and taught skills they can use in their everyday life. This 10-session program can be delivered either in group or individual formats, at any age.
– Cognitive Therapy (Beck, 1987). Individuals suffering from depression are taught cognitive and behavioral skills to help them develop more positive beliefs about themselves, others, and the world. Méndez (1998) argues that therapists working with depressed children should pursue three changes: (1) Learn to value their own feelings; (2) Replace behaviors which generate negative feelings with more appropriate behaviors; and (3) Modify distorted thoughts and inaccurate reasoning. The number of sessions varies between 8 and 16 in patients with mild symptoms. Those with more severe symptoms show improvement after 16 sessions.
– Interpersonal Therapy (Klerman et al., 1984). García and Palazón (2010) identified four typical focal points for tension in depression, related to loss (complicated mourning), conflicts (interpersonal disputes), change (life transitions), and deficits in relations with others (interpersonal deficits), which generate and maintain a depressive state. It uses certain behavioral strategies such as problem solving and social skills training and lasts between 12 and 16 sessions in the most severe cases, and between 3 and 8 sessions in milder cases.
– Cognitive Behavioral Analysis System of Psychotherapy (McCullough, 2000). This therapy combines components of cognitive, behavioral, interpersonal, and psychodynamic therapies. According to McCullough (2003), it is the only therapy developed specifically to treat chronic depression. Patients undergoing this therapy generate more empathic behaviors and identify, change and heal interpersonal patterns related to depression. Patients are recommended to combine the therapy with a regime of antidepressant medication.
– Behavior Therapy/Behavioral Activation (BA) (Martell et al., 2013). Depression prompts sufferers to disengage from their routines and become increasingly isolated. Over time, this isolation exacerbates their depressive symptoms. Depressed individuals lose opportunities to be positively reinforced through pleasant experiences or social activities. The therapy aims to increase patients' chances of being positively reinforced by increasing their activity levels and improving their social relations. The therapy usually lasts between 20 and 24 sessions, with the brief version consisting of between 8 and 15 sessions.
– Problem-Solving Therapy (Nezu et al., 2013). The aim is to enhance patients' personal adjustment to their problems and stress using affective, cognitive, and behavioral strategies. The therapy usually comprises around 12 sessions, although substantial changes are generally observed from the fourth session onwards. This therapy is widely used in primary care. It is an adaptation that is easy to apply in general medicine by personnel working in those contexts, and can be completed in around 6 weeks (Areán, 2000).

The treatments that, according to the American Psychological Association, Society of Clinical Psychology (APA) (2017), have modest research support and could be used with children are as follows:

– Rational Emotive Behavioral Therapy (Ellis, 1994). This short-term, present-focused therapy works on changing the thinking which contributes to emotional and behavioral problems using an active-directive, philosophical and empirical intervention model. Using the A-B-C model (A: events observed by the individual; B: Individual's interpretation of the observed event; C: Emotional consequences of the interpretations made), the aim is to bring about the cognitive restructuring of erroneous thoughts, so as to replace them with more rational ones. The most commonly used techniques are cognitive, behavioral, and emotional.
– Self-System Therapy (Higgins, 1997). Depression occurs as the result of the individual's chronic failure to achieve their established goals. During therapy, patients review their situation, analyze their beliefs and, on the basis of the results, alter their regulation style and move toward a new vision of themselves. Therapy generally consists of between 20 and 25 sessions.
– Short-Term Psychodynamic Therapy (Hilsenroth et al., 2003). The aim of this therapy is to help patients understand that past experiences influence current functioning, and to analyze affect and the expression of emotion. The therapy focuses on the therapeutic relationship, the facilitation of insight, the avoidance of uncomfortable topics and the identification of core conflictual relationship themes. It is usually combined with pharmacological treatment to alleviate depressive episodes.
– Emotion-Focused Therapy (emotion regulation therapy or Greenberg's experiential therapy) (Greenberg, 2004). According to Greenberg et al. (2015), this therapy combines elements of client-based practices (Rogers, 1961), Gestalt therapy (Perls et al., 1951), the theory of emotions and a dialectic-constructivist meta-theory. The aim is to create a safe environment in which the individual's anxiety is reduced, thereby enabling them to confront difficult emotions, raising their awareness of said emotions, exploring their emotional experiences in more depth and identifying maladaptive emotional responses. The therapy is delivered in 8–20 sessions.
– Acceptance and Commitment Therapy (Hayes, 2005). This theory has become increasingly popular over recent years and is the contextual or third-generation therapy that is supported by the largest body of empirical evidence. It is based on a realization of the importance of human language in experience and behavior and aims to change the relationship individuals have with depression and their own thoughts, feelings, memories, and physical sensations that are feared or avoided. Strategies are used to teach patients to decrease avoidance and negative cognitions, and to increase focus on the present. The aim is not to modify the content of the patient's thoughts, but rather to teach them how to change the way they analyze them, since any attempt to correct thoughts may, paradoxically, only serve to intensify them (Hayes, 2005).

Ferdon and Kaslow (2008), for their part, in a theoretical review of the treatment of depression in children and adolescents, concluded that the cognitive-behavioral-therapy-based specific programs of the Penn Prevention program meet the criteria to conduct effective interventions in children with depression. In adolescent depression, the cognitive-behavioral therapy and the Interpersonal Therapy–Adolescent seem to have a well-established efficacy. Weersing et al. (2017), in this same line, state that, although the efficacy of treatments in children is rather weak, cognitive-behavioral therapy is probably the most effective therapy. They also confirm that, in depressed adolescents, cognitive behavioral therapy, and interpersonal psychotherapy are appropriate interventions.

There are other studies also which focus on treatments for depression in childhood. For example, Crowe and McKay (2017) carried out a meta-analysis of the effects of Cognitive Behavioral Therapy (CBT) on children suffering from anxiety and depression, concluding that CBT can be considered an effective treatment for child depression. According to these authors, the majority of protocols for children have been adapted from protocols for adults, and the most common techniques are psychoeducation, self-monitoring, identification of emotions, problem solving, coping skills, and reward plans. Similarly, cognitive strategies include the identification of cognitive errors, also known as cognitive restructuring. In another meta-analysis conducted to analyze the efficacy and acceptability of CBT in cases of child depression, Yang et al. (2017) observed that, in comparison with the control groups that did not receive treatment, the experimental groups showed significant improvement, although they also pointed out that the relevance of this finding was limited due to the small size of the trial groups.

Another study carried out in Saudi Arabia concluded that student counseling in schools may help combat and directly reduce anxiety and depression levels among Saudi children and adolescents (Alotaibi, 2015).

Family-based treatment may also be effective in treating the interpersonal problems and symptoms observed among depressed children. The data indicate that the characteristics of the family environment predict recovery from persistent depression among depressed children (Tompson et al., 2016). In this sense, Tompson et al. (2017) compared the effects of a family-focused treatment for child depression (TCF-DI) with those of individual supportive psychotherapy among children aged 7–14 with depressive disorders. The results revealed that incorporating the family into the therapy resulted in a significant improvement in depressive symptoms, global response, functioning, and social adjustment.

To conclude this section, it can be stated that treatment for depression should be multifactorial and should bear in mind the personal characteristics of the patient, their coping strategy for problems, the type of relationship they have with themselves and the type of relationship they establish with their environment (friends, school, family, etc.). Thus, in order for the individual to attain the highest possible level of psychological wellbeing, attention should focus on both these and other related aspects.

## Conclusions

The present review aims to shed some light on the complex and broad-ranging field of child and adolescent depression, starting with a review of the construct itself and its explanatory theories, before continuing on to analyze existing evaluation instruments, the main prevention programs currently being implemented and the various treatments currently being applied. All these aspects are intrinsically linked: how the concept is defined depends on the explanatory variables upon which said definition is based, and this in turn influences how we measure it and the variables we define as being key elements for its prevention and treatment.

It is interesting to note the low level of specificity of both the construct itself and the explanatory theories offered by child and adolescent psychology, which suggest that child depression can be understood on the basis of the adult version of the pathology. This may well be a basic error in our approach to depression among younger age groups. The fact that universal prevention programs specifically designed for children are obtaining only modest results may indicate that we have perhaps failed to correctly identify the key variables involved in the genesis and maintenance of child and adolescent depression.

The review of current child and adolescent depression prevention programs revealed that the vast majority coincide in adopting a cognitive-behavioral approach, with contents including social skills and problem solving training, emotional education, cognitive restructuring, and strategies for coping with anxiety. These contents are probably included because they are important elements in the treatment of depression, as shown in this review. But if their inclusion is important and effective in the treatment of depression, why do they not seem to be so effective in preventing this pathology? There are probably many factors linked to prevention programs which, in one way or another, influence their efficacy: who implements the program and what prior training they receive; the characteristics of the target group; group dynamics; how sessions are run; how the program is evaluated; and if the proposed goals are really attained (e.g., training in social skills may be key, but perhaps we are not training students correctly). Moreover, in universal prevention programs carried out in schools, the intervention focuses on students themselves rather than adopting a more holistic approach, as recommended by certain authors such as Greenberg et al. (2001). But, if we accept that depression is multifactorial and that risk and protection factors may be found not only in the school environment but also in the family and social contexts, should prevention not also be multifactorial?

There is therefore still much work to be done in order to fully understand child and adolescent depression and its causes, and so design more effective evaluation instruments and prevention and treatment programs. Given the important social and health implications of this disorder, we need to make a concerted effort to further our research in this field.

## Author Contributions

MG designed the study and wrote the protocol. EB and JJ conducted literature review and provided summaries of previous research studies, and wrote the first draft of the manuscript. All authors contributed to and have approved the final manuscript.

### Conflict of Interest Statement

The authors declare that the research was conducted in the absence of any commercial or financial relationships that could be construed as a potential conflict of interest.
